# Liver tumor segmentation in CT volumes using an adversarial densely connected network

**DOI:** 10.1186/s12859-019-3069-x

**Published:** 2019-12-02

**Authors:** Lei Chen, Hong Song, Chi Wang, Yutao Cui, Jian Yang, Xiaohua Hu, Le Zhang

**Affiliations:** 1 School of Computer Science & Technology, Beijing Institute of Technology, 5 South Zhongguancun Street, Beijing, 100081 China; 2 School of Optics and Electronics & Technology, Beijing Institute of Technology, 5 South Zhongguancun Street, Beijing, 100081 China; 3 College of Computing & Informatics, Drexel University, Philadelphia, PA USA; 4 College of Computer Science, Sichuan University, Chengdu, China

**Keywords:** Fully convolutional neural network, CT, Liver segmentation, Liver tumor segmentation

## Abstract

**Background:**

Malignant liver tumor is one of the main causes of human death. In order to help physician better diagnose and make personalized treatment schemes, in clinical practice, it is often necessary to segment and visualize the liver tumor from abdominal computed tomography images. Due to the large number of slices in computed tomography sequence, developing an automatic and reliable segmentation method is very favored by physicians. However, because of the noise existed in the scan sequence and the similar pixel intensity of liver tumors with their surrounding tissues, besides, the size, position and shape of tumors also vary from one patient to another, automatic liver tumor segmentation is still a difficult task.

**Results:**

We perform the proposed algorithm to the Liver Tumor Segmentation Challenge dataset and evaluate the segmentation results. Experimental results reveal that the proposed method achieved an average Dice score of 68.4% for tumor segmentation by using the designed network, and ASD, MSD, VOE and RVD improved from 27.8 to 21, 147 to 124, 0.52 to 0.46 and 0.69 to 0.73, respectively after performing adversarial training strategy, which proved the effectiveness of the proposed method.

**Conclusions:**

The testing results show that the proposed method achieves improved performance, which corroborated the adversarial training based strategy can achieve more accurate and robustness results on liver tumor segmentation task.

## Background

Liver cancer is one of the cancers which has the highest mortality rate in the world. Each year, the morbidity and mortality of liver cancer increase steadily. Computed tomography (CT) is the widely used imaging method for screening, diagnosing, staging, and even prognosis assessment of liver cancer. Segmentation of liver lesions can help physicians diagnose cancer and make appropriate treatment options with more convenient, and can also quickly assess the effectiveness of surgical treatment. However, manually measuring the tumor from a large number of CT slices is very time consuming and relies on the experience of the physician, which is much subjective and susceptible to interference from knowledge differences among different physicians. Therefore, it is very promising to design an objective and accurate scheme for automatic liver tumor segmentation to help physicians better interpret CT images. However, because the number, location, size and shape of liver tumors are significantly different among the patients, additionally, the tumor boundary is always blurred and the contrast between tumor and its surrounding tissues is low, accurate segmentation of liver tumor is still a difficult task.

In order to address this problem, researchers have invested in this study and proposed many methods. During the past few decades, they mainly focused on developing algorithms such as level set, watershed, statistical shape model, region growing, active contour model, threshold processing, graph cuts and traditional machine learning methods that require manually extract tumor features. For example, Zhou et al. [1] proposed a unified level set method (LSM) for liver tumor segmentation. They used local pixel intensity clustering combined with hidden Markov random field to construct a unified LSM. Then, regional information and edge information were used to acquire the tumor contour, so that the problem of edge leakage can be solved. Yan et al. [2] proposed a semi-automatic segmentation method based on watershed transformation. They first placed seed points in the tumor area manually as markers, then watershed transformation was performed to depict and extract the tumor contour in the image. Thus, the density information of tumor can be acquired as threshold to separate liver lesion from its neighborhood tissues. Then, refine the threshold from the segmentation lesions for accurate results. Massoptier et al. [3] proposed an automatic liver tumor segmentation algorithm based on statistical shape model, which used the active contour technique of gradient vector flow to obtain smooth and natural liver tumor segmentation results without the need of interaction. Wong et al. [4] proposed a semi-automatic tumor segmentation method based on region growing method. They first sketch the region of interest of tumor manually and calculate the seed point and feature vector in it, then regional growing algorithm was performed to mark the tumor voxels. Incorporating knowledge based constraints into the growing method ensures the segmented tumor size and shape are within a reasonable range. Yim et al. [5] proposed a segmentation method based on active contour model, which realized liver lesion segmentation by alternating manual initialization and elliptical initialization of the active contour. Through this method, they finally got a good segmentation result. Park et al. [6] proposed a statistical optimal thresholding method for tumor segmentation. They use techniques as maximum posterior decision and binary morphological filtering to segment the liver, then using algorithm such as mixed probability density and minimum total probability error to calculate the optimal threshold. Finally, the segmentation of liver tumor is achieved by performing the calculated threshold. Linguraru et al. [7] proposed a method based on graph cut optimization. They used the shape parameterization method to detect the liver activity contours, which can correct the following abnormal tumor segmentation situation. The tumor segmentation is then realized by shape constrained based graph cut algorithm. In recent years, traditional machine learning based image segmentation methods have played an active role in liver tumor segmentation scenarios. Most of these methods need to manually design tumor feature extraction methods, and developing a model to trained the features, making the model has the ability to identify tumor pixels. Huang et al. [8] proposed a liver tumor segmentation method based on extreme learning machine (ELM), which classifies tumor and non-tumor voxels by training classifiers in stochastic feature subspace sets. The ELM is selected as the basic classifier for model training. To further improve the segmentation performance, voting mechanism is introduced to decide the final classification results of these basic classifiers. Zhou et al. [9] proposed support vector machine (SVM) based method for tumor segmentation. They first trained a SVM model to segment tumor region from a single slice and extracted its contour through morphological operation. Then, the contour is projected to the adjacent slices for next resampling, learning, and classification. Repeating this process until all slices are processed. Most machine learning based methods can achieve better performance than traditional ones, but they are still difficult to learn the accurate tumor feature and susceptible to data fluctuations.

Recently, deep learning has penetrated into a variety of applications and surpassed the state-of-the-art performance in many fields such as image detection, classification, and segmentation [10–15], which also excites us to use this technique in the liver tumor segmentation task. Many researchers have already used deep learning methods to explore the task of liver tumor segmentation. Li et al. [16] proposed a H-DenseUNet which consists of 2D UNet and 3D UNet for liver tumor segmentation. The 2D UNet is used to extract tumor features in a single slice while the 3D UNet is designed to learn the spatial information of tumor between slices. By designing a mixed feature fusion layer to jointly optimize the feature representation between intra- and inter-slice, they finally acquired a satisfactory result in the liver tumor segmentation challenge. Christ et al. [17] proposed a method based on cascading two fully convolutional neural networks (FCN). They first trained an FCN to segment the liver from the abdominal images and used the segmented liver region as input of the second segmentation network, then the tumor segmentation results can be acquired by the second FCN. Finally, they used 3D conditional random field to optimize the tumor segmentation results. Sun [18] et al. proposed a multi-channel fully convolutional network (MC-FCN) based liver tumor segmentation method. They designed an MC-FCN to train contrast-enhanced CT images at different imaging phase due to each phase of the data provides unique information about the pathological features of tumor. Specifically, they performed network training for each phase data in a single channel, and then merged the high-level feature layer in different channels to realize multi-channel training strategy. Experimental results demonstrated the designed network can capture more comprehensive tumor features and improve the model performance in some extent. Yuan et al. [19] developed a hierarchical framework of deep convolution-deconvolution neural network (CDNN) for segmentation of liver tumors. They first trained the CDNN model to segment the liver region from entire abdominal CT sequence, then they performed histogram equalization enhancement on the liver region, and regard it as input of the next CDNN for tumor segmentation. They replaced the loss function by using Jaccard distance during the training process to eliminate the sample reweighting effect and achieved a remarkable segmentation results.

Meantime, generative adversarial network (GAN) has become a more popular emerging technology in many deep learning areas [20–22]. Generator and discriminator are two of its essential components, the responsibility of generator is to make the output of network more similar with the ground truth, while the duty of discriminator is to identify them. Theoretically, the generator output will be adjusted under discriminator’s supervision and move closer to the overall distribution of the ground truth, which can promote the generator’s performance. Motivated by [23], here we proposed an adversarial densely connected network (ADCN) for automatic liver tumor segmentation from CT images. A cascade framework was used during the segmentation process which can utilize the liver regions to reduce false positives. We first used a multi-plane integrated network (MPNet) [24] to segment the liver from the abnormal CT images. Then, a deep fully convolutional neural network (DC-FCN) is designed as the generator to predict the liver tumor from the liver ROI, which is based on convolutional encoder-decoder backbone accompanied with multi-plane convolution, dilated convolution, dense connection and multi-scale feature fusion to enhance the network performance. The training of ADCN is equivalent to jointly optimize the cross-entropy loss with the adversarial training loss which is used to discriminate the output of DC-FCN and ground truth. The discriminator is a convolutional neural network which predicts its input belong to fake (output of DC-FCN) or real (ground truth).

## Methods

### The cascaded framework

**Fig. 1:**
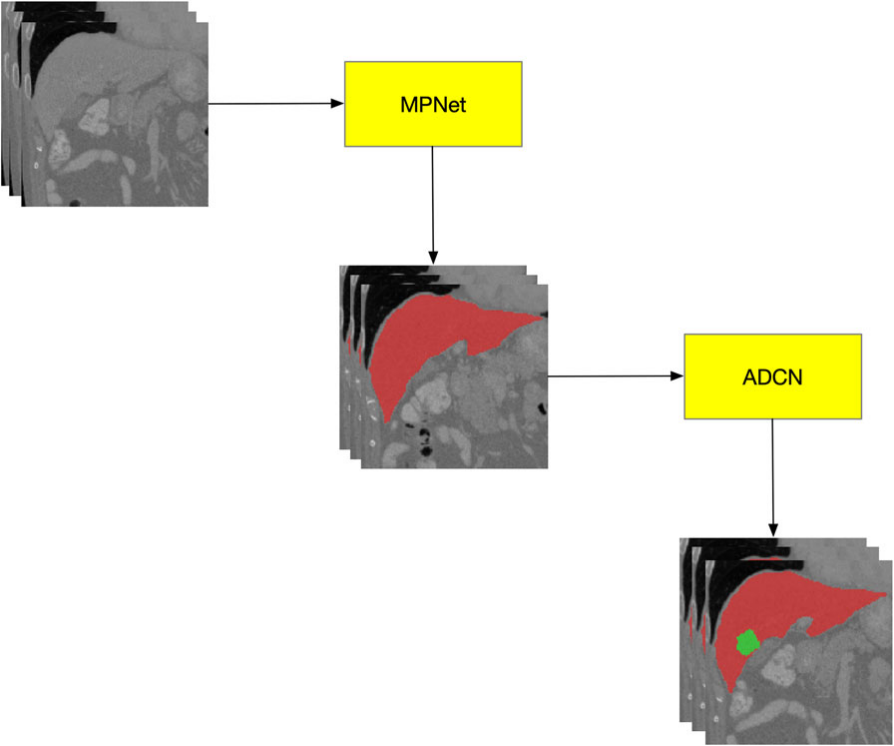
The proposed cascade framework for liver tumor segmentation. Two networks are sequentially used to segment the liver (MPNet) and liver tumor (ADCN)

**Fig. 2:**
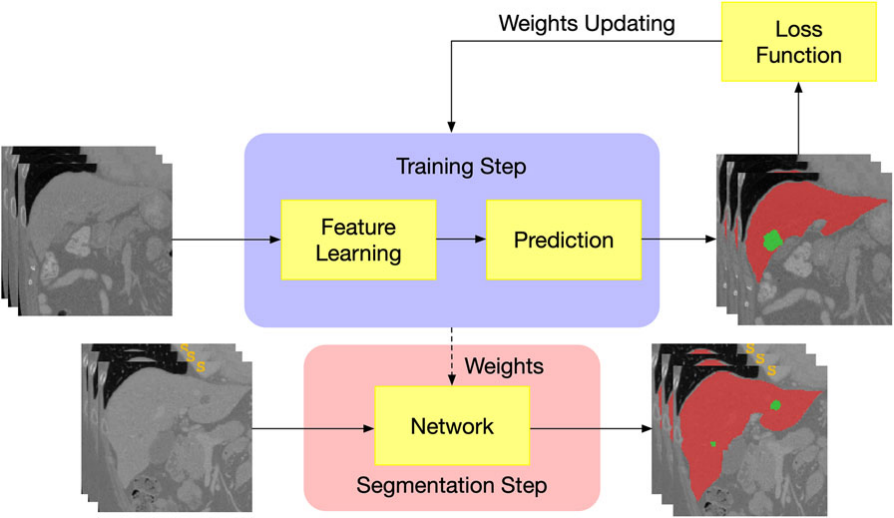
The training and segmentation step of the network construction

Figure 1 shows the proposed cascade framework for liver tumor segmentation. Two networks are sequentially used to segment the liver and liver tumor. The MPNet first segments the liver from abdominal 3D CT volumes, thus we can acquire the accurate liver area. We then use the liver area as the bounding box of ACDN and cropped its input images to train the network for tumor segmentation. The network construction can be summarized as two steps, training the network for weight updating and segmentation using the recording network, as shown in Fig. 2. When training for searching the optimal network parameters of liver or tumor, the bounding boxes are directly selected from the ground truth, while during segmentation step, the bounding boxes of tumor are the segmentation results of liver. Due to the anatomical constraints, this training strategy can help network reduce the false positive rate.

### Multi-plane integrated fully convolutional networks for liver segmentation

The output of an FCN is of a corresponding size with its input,which can be any size. Each voxel input to FCN can acquire a score map for them. In this section, we briefly introduced our previous published method MPNet. Details of the network structure and multi-plane integrate operation can be find in [24]. The main strategy of MPNet separately trained the data from three orthogonal planes (axial, sagittal and coronal) of the input 3D CT volumes. Each network consisted of techniques such as multiple layers of dilated convolution filters, multi-plane convolution filters and residual connections. During segmentation stage, we apply multi-plane integrate technique to fuse the prediction of three networks to generate the final segmentation results. Thus, we can acquire the liver ROI from the abdominal 3D CT volumes.

### Adversarial densely connected network for tumor segmentation

**Fig. 3:**
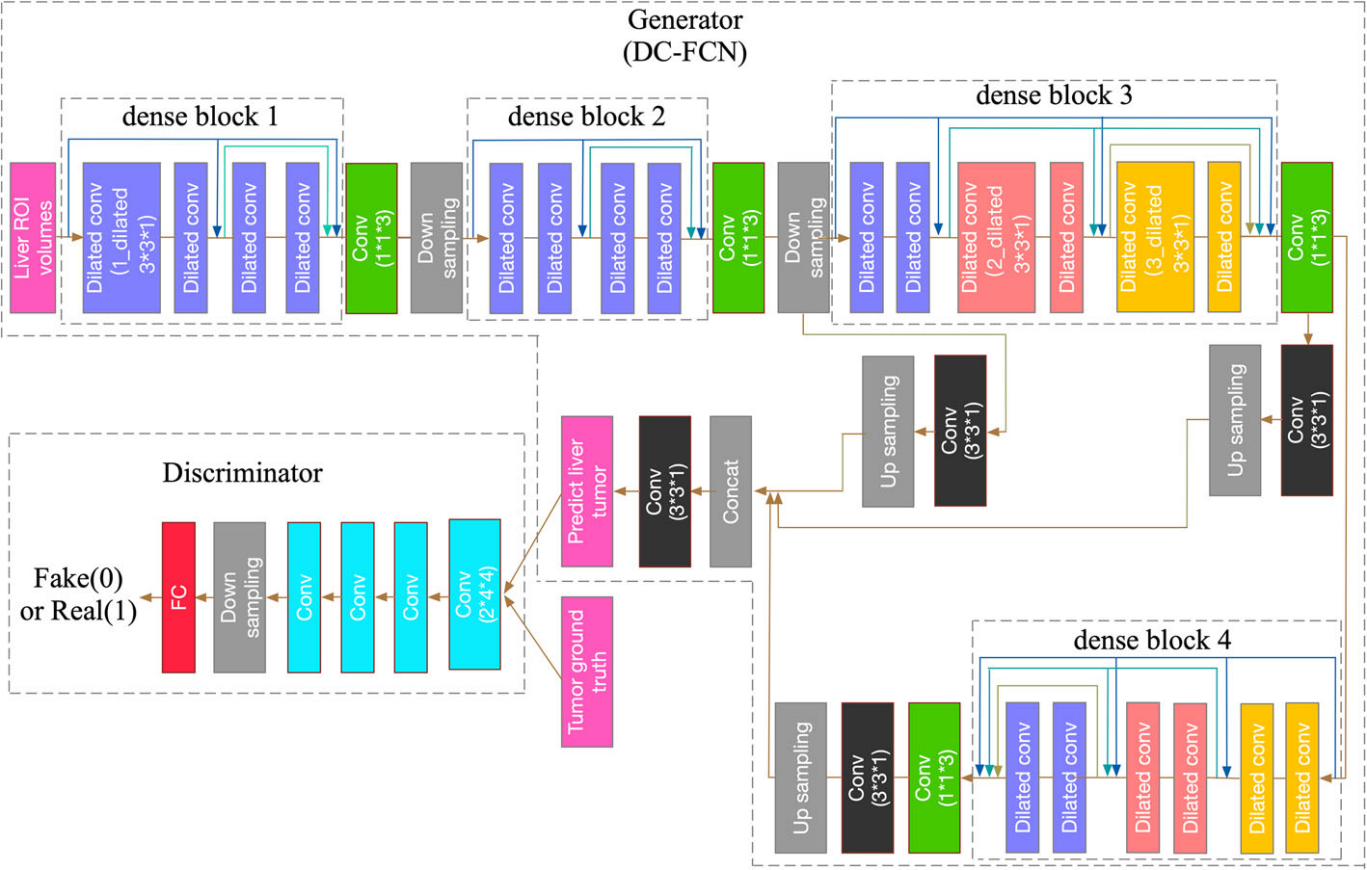
Our adversarial densely connected network with adversarial training, multi-plane convolution, dilated convolution, dense connection block and multi-scale prediction

Through the acquired liver ROI, we can input it into the tumor segmentation network. Due to the proved achievements of the convolutional encoder-decoder structure in image segmentation, we try to improve it for liver tumor segmentation task. In this work, we proposed an adversarial densely connected network with adversarial training strategy, dense connection, dilated convolution and multi-scale feature fusion technology for tumor voxel classification. The structure of ADCN is shown in Fig. 3. We can see that the input of ADCN are 3D CT liver volumes acquired from the MPNet and its output is the probability map that reflects how the voxels belong to tumor. Details of the network are shown in Table 1.

**Table 1:** Detailed parameters of the ADCN model

Layer name	Kernel size	Stride	Padding	Output size
Data	-	-	-	96*96*29*1
Denseblock1(Db1)_Dilated Conv	3*3*1*32	1*1*1	SAME	96*96*29*32
Db1_Dilated Conv	3*3*1*32	1*1*1	SAME	96*96*29*32
Db1_sum	-	-	-	96*96*29*32
Db1_Dilated Conv	3*3*1*32	1*1*1	SAME	96*96*29*32
Db1_Dilated Conv	3*3*1*32	1*1*1	SAME	96*96*29*32
Db1_sum	-	-	-	96*96*29*32
Fuse	1*1*3	1*1*1	VALID	96*96*27*32
Down sampling	3*3*1	2*2*1	SAME	48*48*27*32
Denseblock2(Db2)_Dilated Conv	3*3*1*32	1*1*1	SAME	48*48*27*32
Db2_Dilated Conv	3*3*1*32	1*1*1	SAME	48*48*27*32
Db2_sum	-	-	-	48*48*27*32
Db2_Dilated Conv	3*3*1*32	1*1*1	SAME	48*48*27*32
Db2_Dilated Conv	3*3*1*32	1*1*1	SAME	48*48*27*32
Db2_sum	-	-	-	48*48*27*32
Fuse	1*1*3	1*1*1	VALID	48*48*25*32
Down sampling	3*3*1	2*2*1	SAME	48*48*25*32
Denseblock3(Db3)_Dilated Conv	3*3*1*32	1*1*1	SAME	24*24*25*32
Db3_Dilated Conv	3*3*1*32	1*1*1	SAME	24*24*25*32
Db3_sum	-	-	-	24*24*25*32
Db3_Dilated Conv	3*3*1*32	1*1*1	SAME	24*24*25*32
Db3_Dilated Conv	3*3*1*32	1*1*1	SAME	24*24*25*32
Db3_sum	-	-	-	24*24*25*32
Db3_Dilated Conv	3*3*1*32	1*1*1	SAME	24*24*25*32
Db3_Dilated Conv	3*3*1*32	1*1*1	SAME	24*24*25*32
Db3_sum	-	-	-	24*24*25*32
Fuse	1*1*3	1*1*1	VALID	24*24*23*32
Denseblock4(Db4)_Dilated Conv	3*3*1*32	1*1*1	SAME	24*24*23*32
Db4_Dilated Conv	3*3*1*32	1*1*1	SAME	24*24*23*32
Db4_sum	-	-	-	24*24*23*32
Db4_Dilated Conv	3*3*1*32	1*1*1	SAME	24*24*23*32
Db4_Dilated Conv	3*3*1*32	1*1*1	SAME	24*24*23*32
Db4_sum	-	-	-	24*24*23*32
Db4_Dilated Conv	3*3*1*32	1*1*1	SAME	24*24*23*32
Db4_Dilated Conv	3*3*1*32	1*1*1	SAME	24*24*23*32
Db4_sum	-	-	-	24*24*23*32
Fuse	1*1*3	1*1*1	VALID	24*24*23*32
Up sampling1	-	-	-	96*96*21*2
Up sampling2	-	-	-	96*96*21*4
Up sampling3	-	-	-	96*96*21*8
Concat	-	-	-	96*96*21*14
Conv	3*3*1*2	1*1*1	SAME	96*96*21*2

The backbone of ADCN follows the concept of convolutional encoder-decoder. For training 3D network, we must consider the trade-off between the memory consumption and receptive field. Using small receptive field can only capture local features while large receptive field can capture more global information. However, using large 3D receptive field need input a lot of 3D patches for training, which will cost much computing memory. Here because our DC-FCN uses a stake of 3D slices as input, we set a large 2D receptive field with size of 217*217 to learn the tumor features intra-slice and a receptive field with size of 12 to capture the inter-slice tumor information. Then, we designed a 3×3×1 intra-slice kernel and a 1×1×3 inter-slice kernel to realize the function of 3×3×3 kernel. A batch normalization layer and parametric rectified linear units (PReLU) activation layer [25] was conventional attached behind each convolution layer. The total amount of intra-slice and inter-slice convolution layer of DC-FCN are 20 and 4, respectively. In order to guarantee the image resolution and loss of tumor details, we just use two downsampling layers in the network. To make the receptive field of intra-slice larger, here we use dilated conventional with different parameter as kernel in our network, which is shown in blue, red and orange blocks in Fig. 3.

Here we converted residual connection [26] to dense connection [27] which connects each layer to every other layer for effective extracting tumor features in deep networks. Our ADCN has four dense blocks, which the first two blocks consists of 4 intra-slice convolution layers and the last two blocks consist of 6 intra-slice convolution layers. For each layer, its input are the feature maps output from previous layers, and its output are used as inputs to subsequent layers, which helps the current layer to learn information with reference to the previous input. Introducing dense connection can help information reuse, smooth feature propagation and promote the network convergence.

**Fig. 4:**
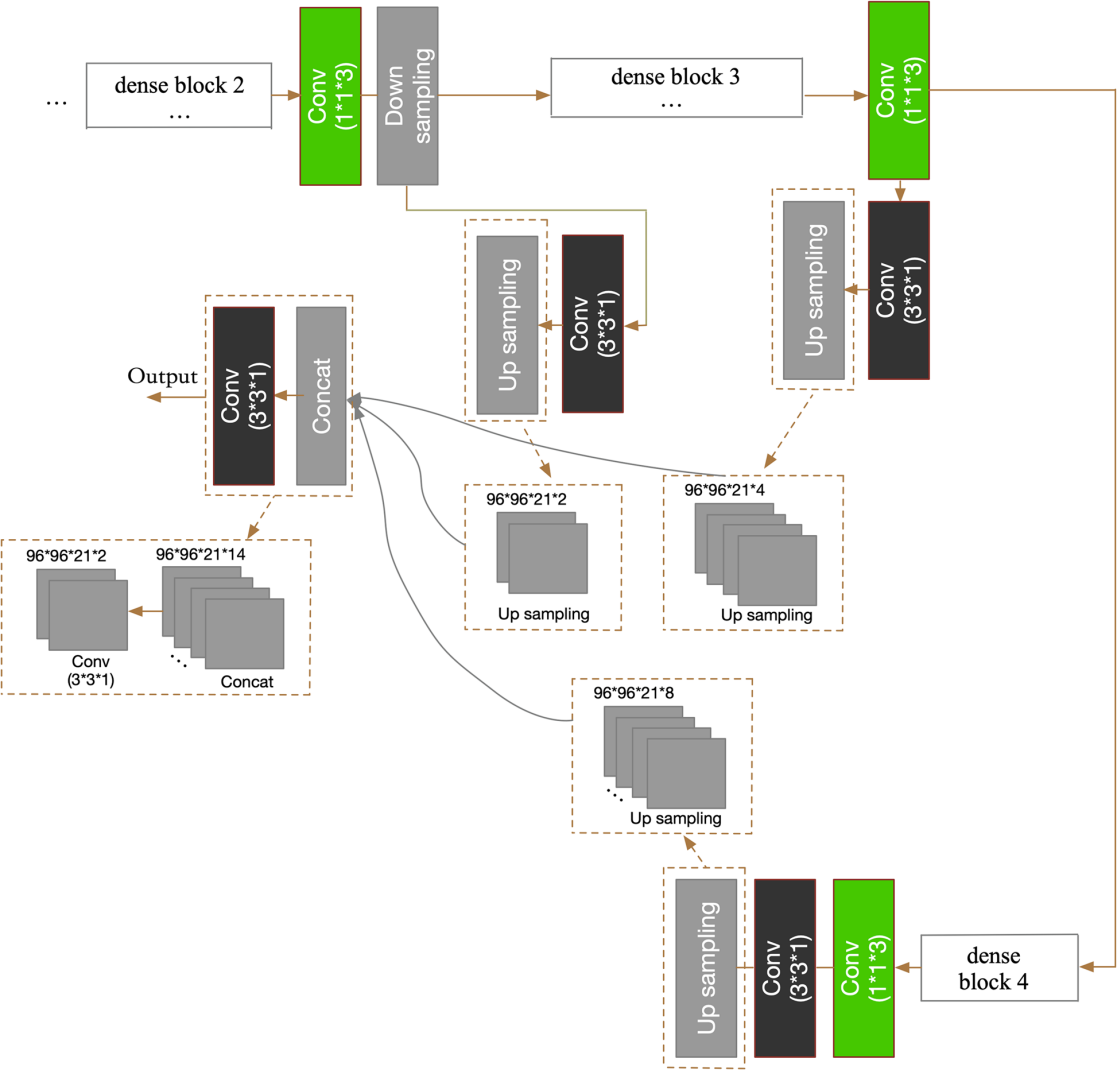
The multi-scale prediction process of our DC-FCN

In our DC-FCN, the shallow convolution layers try to capture low-level and local tumor features while the deep convolution layers try to learn advanced and global feature representation. Inspired by [28] of fusing prediction results from multiple scales, here we combined the tumor feature at three different network depths to realize multi-scale prediction as shown in Fig. 4. Specifically, we acquired the different intermediate prediction results by applying 3×3×1 convolution layer after corresponding layer as shown in black blocks in Fig. 4, and then upsampling them directly to the resolution equal to the initial input. Then the final score map of tumor segmentation is acquired by sending the concatenation result of the above feature maps to an extra 3×3×1 convolution layer. The output layer has 2 channels which represent the tumor and non-tumor two segmentation classes. Details of the network structure are shown in Table 1.

To further improve the performance of DC-FCN, we try to use the popular idea of introducing adversarial training strategy [29] in our network. Here we designed a deep convolutional generative adversarial network (DC-GAN) [30] based method to boost the tumor segmentation results of DC-FCN. The generator and discriminator structure of the designed network can be seen in Fig. 3. The generator is DC-FCN and discriminator is a CNN consists of five convolution layers with kernel size of 2×4×4, a down sampling layer with size of 2×6×6 and a fully connected layer. Each convolution layer is followed by a batch normalization and Leaky ReLU layer for promoting gradient propagation. The high-level information of tumor appearance can be utilized by adversarial network to better identify the output of DC-FCN and the ground truth. In training stage, there will be an adversarial loss function provided by the network, which aims to make the output of DC-FCN more similar to the ground truth by optimizing the generator’s parameters to help it predicting more accurate tumor results. We used the adversarial loss for training the network. For the discriminator *D*(*Y*;*w*_*D*_), where *w*_*D*_ is its weights and we set the tumor ground truth *Y*_*gt*_as 1 and the prediction *Y*_*p*_=*G*(*X*,*w*_*G*_) as 0, where *G* is the generator, *X* is the input liver data and *w*_*G*_ is the weights of generator. During adversarial training, we used the objective function as follows,

1 $$ {{\begin{aligned}L_{D} &=-{E_{y/p_{gt}}}log(D(y;w_{D}))-{E_{{y^{\prime}}/p_{pred}}}log(1-D({y^{\prime}};w_{D}))\\ &=-{E_{y/p_{gt}}}log(D(y;w_{D}))-{E_{x/p_{data}}}log(1-D(G(x;w_{G});w_{D})) \end{aligned}}}  $$

Where *y*^′^=*D*(*G*(*x*;*w*_*G*_) is the output of *G*, *p*_*gt*_ represents that the output is belong to ground truth, *p*_*pred*_ represents that the output is belong to prediction. When training discriminator *D*, the gradient of discriminator loss *L*_*D*_ will be propagated back to optimize the weights of the generator (DC-FCN), which the current generator loss *L*_*G*_ consists of the following two parts as shown in Eq. 2, where *p*_*data*_ represents to sample a mini-batch from training images and *λ* is a variable. The first part is the tumor content loss *L*_*seg*_, which is calculated by performing cross entropy to the segmentation results and ground truth. And the second part aims to confuse the discriminator with the output of generator more similar to the ground truth.

2 $$ {{\begin{aligned} L_{G}&= {E_{y/p_{pred},{y^{\prime}}\! /p_{gt}}}[\!L_{seg}(y,{y^{\prime}})]\! -\lambda {E_{y/p_{pred}}}log(1-D(y;w_{D})) \\ &={E_{y/p_{pred},{y^{\prime}}\! /p_{gt}}}[\! L_{seg}(y,{y^{\prime}})]\! -\lambda {E_{x/p_{data}}}log(1\! -\! D(x;w_{G});w_{G}) \end{aligned}}}  $$

Alternatively training the generator and discriminator until the output of DC-FCN can not be easily discriminated with tumor ground truth anymore. Then we drop out the discriminator to end the adversarial training process, which the performance of the generator has already improved to predict better tumor results.

## Results and discussion

The 2017 liver tumor segmentation (LiTS) Challenge dataset was used to train the MPNet, DC-FCN and ADCN models. Then, the liver tumors were segmented using the trained models.

### Data and implementation details

There are a total of 131 abdominal 3D CT scans in 2017 LiTS Challenge dataset, which the slices have the size of 512x512 in axial plane. We selected 121 scans to train our model and the remaining 10 scans to test the model performance. Due to the data is collected by various scanning protocols from European hospitals, which lead to a non-uniform slice spacing and large resolution difference. We first re-sampled all the scans to an isotropic resolution of 1*mm*^3^. The manual segmentation of liver and its tumors were performed by experienced experts as ground truth.

As the experimental environment, here we adopted TensorFlow with NiftyNet [31] to implement our models, which used Adaptive Moment Estimation (Adam) [32] with initial learning rate of 1×10^−3^, weight decay of 1×10^−7^ for training. We set the maximal iteration of 20k in all the experiments. The training patch size for liver was set to 256×256×19 and for tumor is 96×96×29. The Dice loss function [33] was selected to train our network. A computer with the Linux Ubuntu 16.04 LST 64-bit operating system cooperated with a 32 GB Intel i7 3.4 GHz CPU and a NVIDIA GeForce GTX 1080 Ti graphics card with 12GB memory was used in our experiment.

### Quantitative evaluations criteria

We provided quantitative measurements including Dice score, average symmetric surface distance (ASD), mean square symmetric surface distance (MSD), relative volume difference (RVD) and volumetric overlap error (VOE) to evaluate the effectiveness of our proposed model. Here we use *S* to represent the segmented liver/liver tumor region, and *G* is the ground truth, *r* is a point of arbitrary surface voxel, *d* is the Euclidean distance, *R*(*S*) and *R*(*G*) are the areas of liver/tumor region in *S* and *G*, respectively.

The Dice score can be evaluated as follows,

3 $$\begin{array}{@{}rcl@{}} Dice(S,G)= \frac{2\left|S \bigcap G\right|}{\left|S\right|+\left|G\right|} \end{array} $$

Where the score is between [0,1], an ideal segmentation is expressed as the Dice score close to 1.

The ASD is related to the surface voxels of *S* and *G*, by calculating the *d* of surface voxel *S* to the closest surface voxel *G* and repeat the same process from the surface voxel *G* to those of surface voxel *S*. The ASD can be calculated as the mean value of all distances, which can be represented in Eq. 4, for which a smaller value represents a better segmentation result.

4 $$ {{}{\begin{aligned} ASD(S,G) &=\frac{1}{\left|R(S)\right|+\left|R(G)\right|}\left(\sum_{{r_{S}}\in R(S)}d(r_{S}, R(G)) \right.\\ &\quad \left. + \sum_{{r_{G}}\in R(G)} d(r_{G}, R(S))\right) \end{aligned}}}  $$

The calculating of MSD is similar with ASD, which is also related to the surface voxels of *S* and *G* but need to calculate the mean square error as follows,

5 $$ {\begin{aligned} &MSD(S,G)\\&=max{\left({max}_{{r_{S}}\in R(S)}{d^{2}}\left(r_{S}, R(G)\right), {max}_{{r_{G}}\in R(G)}{d^{2}}(r_{G}, R(S))\right)} \end{aligned}}  $$

The RVD can be represented as follows, An RVD of 0 represents that the region of *S* and *G* are identical.

6 $$\begin{array}{@{}rcl@{}} RVD(S,G)=\frac{\left|G\right|-\left|S\right|}{\left|S\right|} \end{array} $$

And the VOE can be calculated as follows, the larger VOE value represents the worse segmentation performance.

7 $$\begin{array}{@{}rcl@{}} VOE(S,G)= 1-\frac{\left|S \bigcap G\right|}{\left|S\bigcup G\right|} \end{array} $$

### Liver segmentation results

**Fig. 5:**
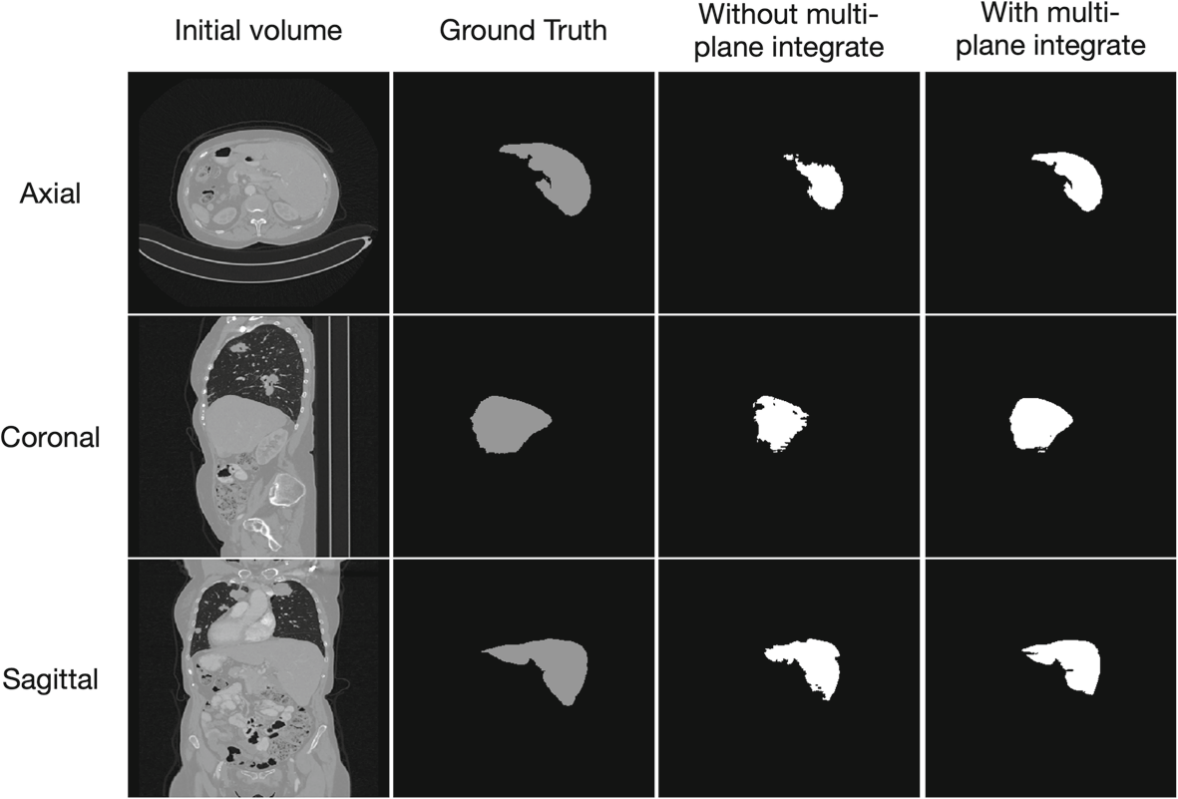
The liver segmentation results in three orthogonal views

The segmentation results of liver from testing images are shown in Fig. 5, we compared the proposed method with the network which is only trained in axial plane. It can be seen easily of the under segmentation phenomenon without using the multi-plane integrate training method, which proved the effectiveness of our MPNet in liver tumor segmentation tasks. For quantitative evaluation, the MPNet has achieved an average Dice score of 0.967, VOE of 0.063, ASD of 1.32 and MSD of 29.9 for liver segmentation, respectively, while the model trained only in axial plane has achieved a Dice score of 0.685, VOE of 0.438, ASD of 25 and MSD of 92.3, which further proved that using multi-plane integrate prediction can help model utilize more robust liver features for segmentation. It took us about 97 h to train the MPNet due to the need of training three separate networks. However, during testing stage, the time to deal with one case is related to its slices number, which is ranging from 13 s to 85 s for network without multi-plane integrate, and 30 s to 250 s for MPNet.

### Tumor segmentation results

**Fig. 6:**
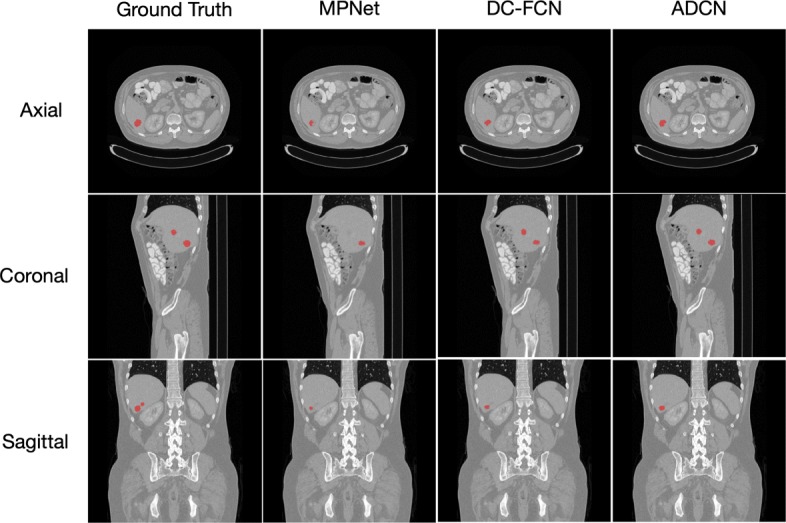
The liver tumor segmentation results using different networks from a training image

**Fig. 7:**
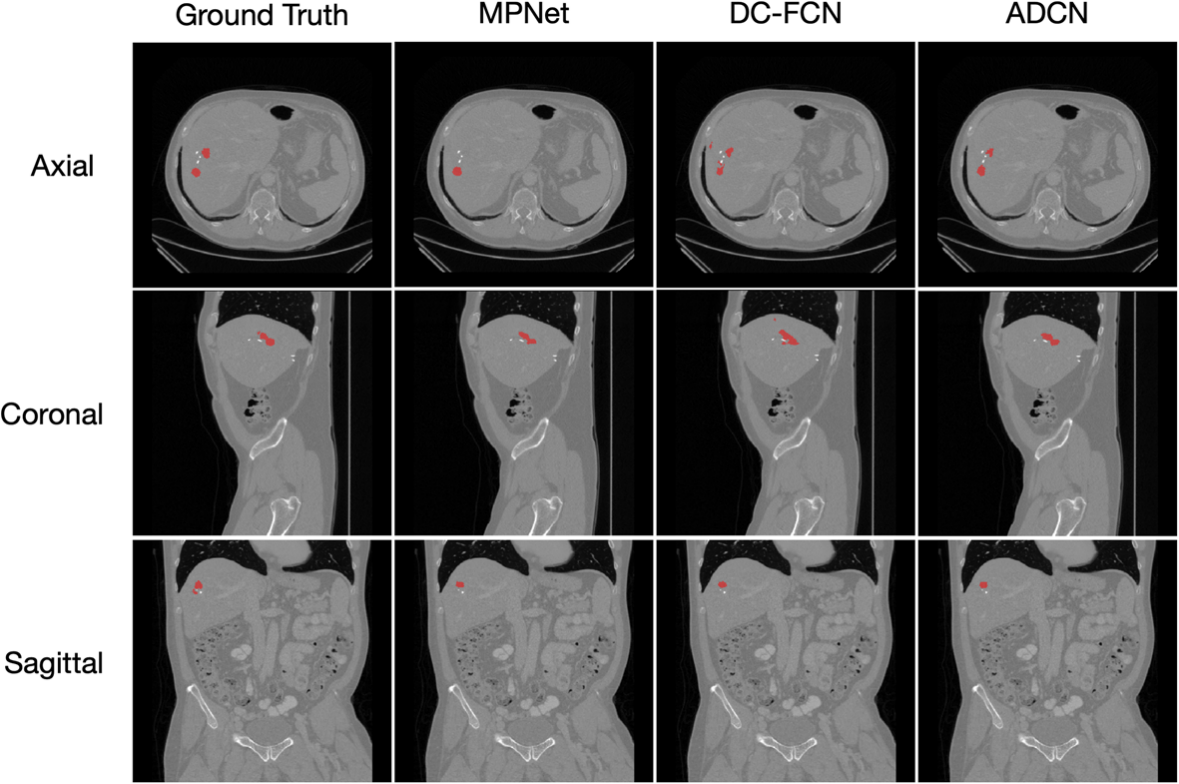
Example of tumor segmentation results from a testing image

Figures 6 and 7 show the tumor segmentation results from training and testing images, respectively. The red colors represent the liver tumor. We compared the results generated by ground truth with residual based MPNet, densely connected based DC-FCN and adversarial training based ADCN. To visualize the results simplicity caused by the network differences, here we only trained the network in axial plane. In Fig. 6, results provided by MPNet, DC-FCN and ADCN are presented in the second, third and forth columns, respectively, which we can see that for the MPNet segmentation results, residual connection can discriminate the tumor in some extent, but miss a part of tissues which should belong to tumor, while the DC-FCN can predict more accurate segmentation results with the help of dense connection, but still have the under segmentation problem compared with the results generated by ADCN, which was benefited from the adversarial training strategy and can identify more voxels that belong to tumor. In Fig. 7, the achieved results of a testing image show similar appearance with the training one. However, it can be seen that the segmentation of liver tumor produced by ADCN is more accurate. Although the segmentation results provide by ADCN still have some unsegmented tumor tissues, it’s already have a significant improvement with the other two methods, which proves the effectiveness of the proposed algorithm.

**Fig. 8:**
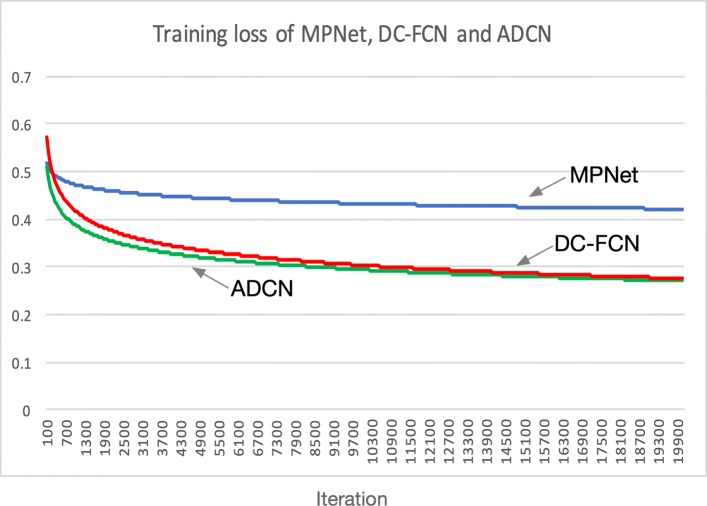
Training loss of the three networks

Results of performing power function to the loss values of MPNet, DC-FCN and ADCN are shown in Fig. 8, it is observed that all the three network training costs about 18k iterations to converge and the overall training loss of ADCN is lower than DC-FCN by using adversarial training strategy, and the loss of DC-FCN is lower than MPNet by using dense connection technology, the advantage of combining these two techniques has further proved the network performance.

**Fig. 9:**
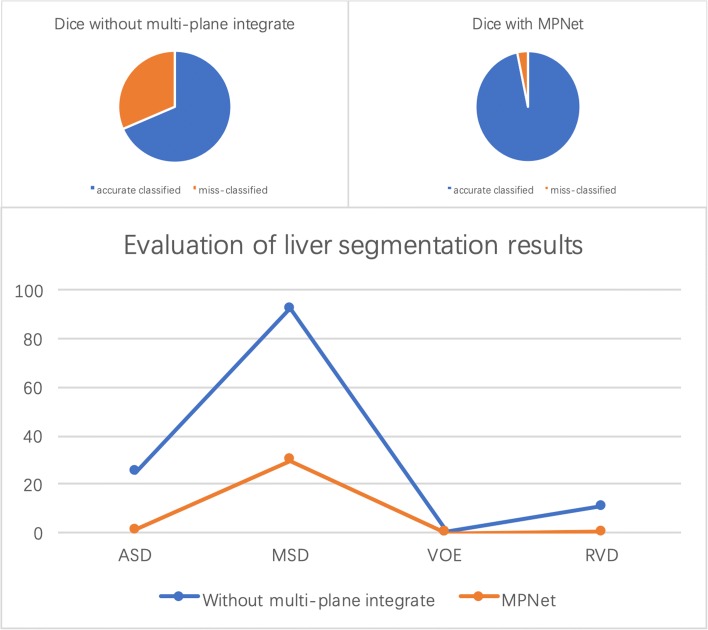
Quantitative evaluation results for liver segmentation

**Fig. 10:**
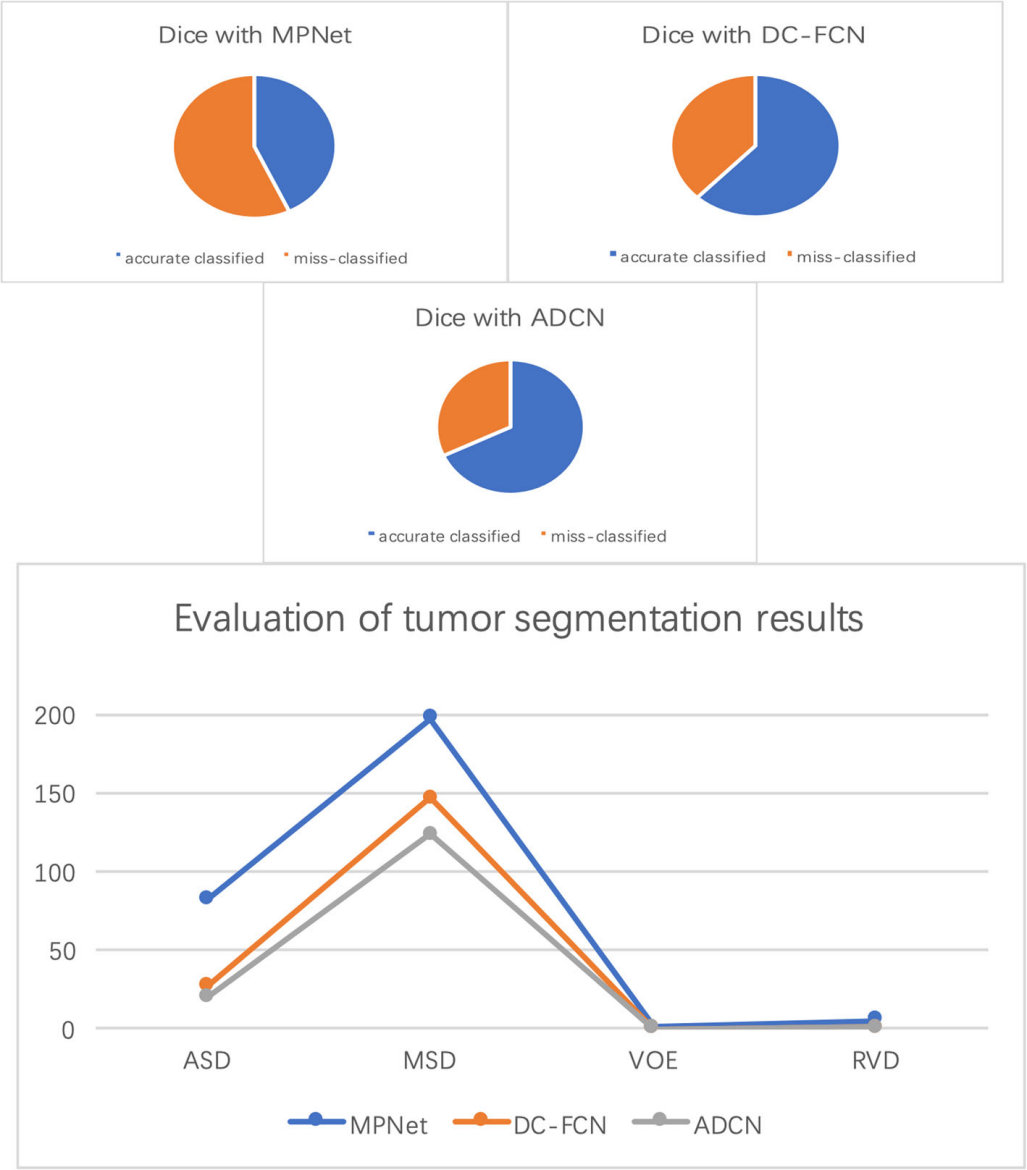
Quantitative evaluation results for tumor segmentation

Figures 9 and 10 show quantitative evaluation results for liver and liver tumor segmentation on the testing set. It can be seen that the proposed method achieved average Dice scores of 0.684 and 0.967, ASD of 25 and 1.32, MSD of 92.3 and 29.9, VOE of 0.438 and 0.063, RVD of 11 and 0.2 by using network without multi-plane integrate and MPNet in liver segmentation task, while for tumor segmentation, our MPNet, DC-FCN and ADCN have achieved average Dice scores of 0.427, 0.616 and 0.684 with the ASD improved from 82.6, 27.8 to 21 and MSD improved from 198, 147 to 124, VOE improved from 0.68, 0.52 to 0.46 and RVD changed from 5.26, 0.69 to 0.73, respectively. We can see that after combining dense connection and adversarial training strategy, evaluation results on both qualitative and quantitative can be improved in some extent. Compared the performance with other evaluation index, although the RVD value have increased 0.04 after introducing adversarial training, it will not affect the overall performance of segmentation task, which further proved the effectiveness of the proposed strategy.

The benefit of using cascade framework can help reduce the complexity of designing tumor segmentation network and make it easier to train. It can also help reduce false positives since ADCN works on the region segmented by MPNet. What’s more, our network can use the spatial constraints provided by anatomical structures of the liver and tumor, which the segmented liver mask restrict the liver tumor inside the liver region. However, this kind of training strategy is always not end-to-end, which has a defect of longer training time compared with other methods using similar network structure. But as far as we consider, the training time is not an important metric in liver tumor segmentation task.

Examples of the result show that our method acquired a satisfied performance for both liver and tumor segmentation tasks. The multi-plane integrate technology helps network get more higher accuracy, which is mainly benefited from utilizing the 3D contextual information in three orthogonal planes. And the dense connection combined with adversarial training strategy further improve the model performance because the deep reuse of spatial information from tumor sequence and its global appearance.

## Conclusions

In this paper, we developed a cascaded adversarial training system to segment liver tumors from abdominal CT volumes. The liver tumor segmentation challenge was divided into a two cascade binary segmentation tasks and we designed two networks to segment the liver and liver tumor, respectively. Specifically, we first used our previous method named multi-plane integrate network to segment the liver tissue from 3D CT abdominal volumes. Then we extracted the tumors in the liver region by develop a deep 3D densely connected fully convolutional neural network with adversarial training strategy. Our networks use a multi-plane convolution operation, which balanced the computing memory consumption and receptive field. We also introduced dense connection to capture more accurate tumor features followed with multi-scale feature fusion technique to reduce the miss segmented results. Adversarial training strategy is used to minimize the output of network with ground truth, which further boosts the final segmentation result. Experimental results show that our method achieved a best Dice score of 68.4% for tumor segmentation, and ASD, MSD, VOE and RVD improved from 27.8 to 21, 147 to 124, 0.52 to 0.46 and 0.69 to 0.73, respectively.

## Data Availability

The data used in our paper were available through https://competitions.codalab.org

## Abbreviations

Adam: Adaptive moment estimation
ADCN: Adversarial densely connected network
ASD: Average symmetric surface distance
CDNN: Convolution-deconvolution neural network
CNN: Convolutional neural network
CT: Computed tomography
DC-FCN: Deep fully convolutional neural network
DC-GAN: Deep convolutional generative adversarial network
ELM: Extreme learning machine
FCN: Fully convolutional neural network
GAN: Generative adversarial network
LiTS: liver tumor segmentation
LSM: Level set method
MC-FCN: Multi-channel fully convolutional network
MPNet: Multi-plane integrated network
MSD: Mean square symmetric surface distance
PReLU: Parametric rectified linear units
ROI: Region of interest
RVD: Relative volume difference
SVM: Support vector machine
VOE: Volumetric overlap error

## References

[1] Zheng Zhou, Zhang Xuechang, Xu Huafei, Liang Wang, Zheng Siming, Shi Yueding (2018). A Unified Level Set Framework Combining Hybrid Algorithms for Liver and Liver Tumor Segmentation in CT Images. BioMed Research International.

[2] Yan J, Schwartz LH, Zhao B (2015). Semiautomatic segmentation of liver metastases on volumetric ct images. Med Phys.

[3] Massoptier L, Casciaro S (2008). A new fully automatic and robust algorithm for fast segmentation of liver tissue and tumors from ct scans. Eur Radiol.

[4] Wong D, Liu J, Fengshou Y, Tian Q, Xiong W, Zhou J, Qi Y, Han T, Venkatesh S, Wang S-c (2008). A semi-automated method for liver tumor segmentation based on 2d region growing with knowledge-based constraints. MICCAI Workshop, vol. 41.

[5] Yim PJ, Foran DJ. Volumetry of hepatic metastases in computed tomography using the watershed and active contour algorithms. In: 16th IEEE Symposium Computer-Based Medical Systems, 2003. Proceedings. IEEE: 2003. p. 329–35. 10.1109/cbms.2003.1212810.

[6] Park Seung-Jin, Seo Kyung-Sik, Park Jong-An (2005). Automatic Hepatic Tumor Segmentation Using Statistical Optimal Threshold. Lecture Notes in Computer Science.

[7] Linguraru Marius George, Richbourg William J., Watt Jeremy M., Pamulapati Vivek, Summers Ronald M. (2012). Liver and Tumor Segmentation and Analysis from CT of Diseased Patients via a Generic Affine Invariant Shape Parameterization and Graph Cuts. Lecture Notes in Computer Science.

[8] Huang W, Yang Y, Lin Z, Huang G. -B., Zhou J, Duan Y, Xiong W. Random feature subspace ensemble based extreme learning machine for liver tumor detection and segmentation. In: 2014 36th Annual International Conference of the IEEE Engineering in Medicine and Biology Society. IEEE: 2014. p. 4675–8. 10.1109/embc.2014.6944667. 25571035

[9] Zhou J, Xiong W, Tian Q, Qi Y, Liu J, Leow WK, Han T, Venkatesh SK, Wang S-c (2008). Semi-automatic segmentation of 3d liver tumors from ct scans using voxel classification and propagational learning. MICCAI Workshop, vol. 41.

[10] Krizhevsky Alex, Sutskever Ilya, Hinton Geoffrey E. (2017). ImageNet classification with deep convolutional neural networks. Communications of the ACM.

[11] Xu Y, Du J, Dai L-R, Lee C-H (2014). An experimental study on speech enhancement based on deep neural networks. IEEE Signal Process Lett.

[12] Yu L, Chen H, Dou Q, Qin J, Heng P-A (2017). Automated melanoma recognition in dermoscopy images via very deep residual networks. IEEE Trans Med Imaging.

[13] Yu L, Chen H, Dou Q, Qin J, Heng P-A (2017). Automated melanoma recognition in dermoscopy images via very deep residual networks. IEEE Trans Med Imaging.

[14] Shin H. -C., Roth HR, Gao M, Lu L, Xu Z, Nogues I, Yao J, Mollura D, Summers RM (2016). Deep convolutional neural networks for computer-aided detection: Cnn architectures, dataset characteristics and transfer learning. IEEE Trans Med Imaging.

[15] Havaei M, Davy A, Warde-Farley D, Biard A, Courville A, Bengio Y, Pal C, Jodoin P-M, Larochelle H (2017). Brain tumor segmentation with deep neural networks. Med Image Anal.

[16] Li X, Chen H, Qi X, Dou Q, Fu C-W, Heng P-A (2018). H-denseunet: Hybrid densely connected unet for liver and tumor segmentation from ct volumes. IEEE Trans Med Imaging.

[17] Christ PF, Ettlinger F, Grün F, Elshaera MEA, Lipkova J, Schlecht S, Ahmaddy F, Tatavarty S, Bickel M, Bilic P, et al.Automatic liver and tumor segmentation of ct and mri volumes using cascaded fully convolutional neural networks. 2017. arXiv preprint arXiv:1702.05970.

[18] Sun C, Guo S, Zhang H, Li J, Chen M, Ma S, Jin L, Liu X, Li X, Qian X (2017). Automatic segmentation of liver tumors from multiphase contrast-enhanced ct images based on fcns. Artif Intell Med.

[19] Yuan Y. Hierarchical convolutional-deconvolutional neural networks for automatic liver and tumor segmentation. 2017. arXiv preprint arXiv:1710.04540.

[20] Ledig C, Theis L, Huszár F, Caballero J, Cunningham A, Acosta A, Aitken A, Tejani A, Totz J, Wang Z, et al.Photo-realistic single image super-resolution using a generative adversarial network. In: Proceedings of the IEEE Conference on Computer Vision and Pattern Recognition: 2017. p. 4681–90. 10.1109/cvpr.2017.19.

[21] Nie Dong, Trullo Roger, Lian Jun, Petitjean Caroline, Ruan Su, Wang Qian, Shen Dinggang (2017). Medical Image Synthesis with Context-Aware Generative Adversarial Networks. Medical Image Computing and Computer Assisted Intervention − MICCAI 2017.

[22] Shrivastava A, Pfister T, Tuzel O, Susskind J, Wang W, Webb R. Learning from simulated and unsupervised images through adversarial training. In: Proceedings of the IEEE Conference on Computer Vision and Pattern Recognition: 2017. p. 2107–16. 10.1109/cvpr.2017.241.

[23] Luc P, Couprie C, Chintala S, Verbeek J. Semantic segmentation using adversarial networks. 2016. arXiv preprint arXiv:1611.08408.

[24] Wang C, Song H, Chen L, Li Q, Yang J, Hu XT, Zhang L. Automatic liver segmentation using multi-plane integrated fully convolutional neural networks. In: 2018 IEEE International Conference on Bioinformatics and Biomedicine (BIBM). IEEE: 2018. p. 1–6. 10.1109/bibm.2018.8621257.

[25] He K, Zhang X, Ren S, Sun J. Delving deep into rectifiers: Surpassing human-level performance on imagenet classification. In: Proceedings of the IEEE International Conference on Computer Vision: 2015. p. 1026–34. 10.1109/iccv.2015.123.

[26] He K, Zhang X, Ren S, Sun J. Deep residual learning for image recognition. In: Proceedings of the IEEE Conference on Computer Vision and Pattern Recognition: 2016. p. 770–8. 10.1109/cvpr.2016.90.

[27] Huang G, Liu Z, Van Der Maaten L, Weinberger KQ. Densely connected convolutional networks. In: Proceedings of the IEEE Conference on Computer Vision and Pattern Recognition: 2017. p. 4700–8. 10.1109/cvpr.2017.243.

[28] Fidon Lucas, Li Wenqi, Garcia-Peraza-Herrera Luis C., Ekanayake Jinendra, Kitchen Neil, Ourselin Sébastien, Vercauteren Tom (2018). Generalised Wasserstein Dice Score for Imbalanced Multi-class Segmentation Using Holistic Convolutional Networks. Brainlesion: Glioma, Multiple Sclerosis, Stroke and Traumatic Brain Injuries.

[29] Ganin Y, Ustinova E, Ajakan H, Germain P, Larochelle H, Laviolette F, Marchand M, Lempitsky V (2016). Domain-adversarial training of neural networks. J Mach Learn Res.

[30] Radford A, Metz L, Chintala S. Unsupervised representation learning with deep convolutional generative adversarial networks. 2015. arXiv preprint arXiv:1511.06434.

[31] Gibson E, Li W, Sudre C, Fidon L, Shakir DI, Wang G, Eaton-Rosen Z, Gray R, Doel T, Hu Y (2018). Niftynet: a deep-learning platform for medical imaging. Comput Methods Prog Biomed.

[32] Kingma DP, Ba J. Adam: A method for stochastic optimization. 2014. arXiv preprint arXiv:1412.6980.

[33] Milletari F, Navab N, Ahmadi S-A. V-net: Fully convolutional neural networks for volumetric medical image segmentation. In: 2016 Fourth International Conference on 3D Vision (3DV). IEEE: 2016. p. 565–71. 10.1109/3dv.2016.79.

